# Surgical Imperatives in Benign Follicular Nodular Disease: Redefining Malignancy Risk and the Role of Total Thyroidectomy

**DOI:** 10.1155/bmri/6651666

**Published:** 2026-03-10

**Authors:** Azhy Muhammed Dewana

**Affiliations:** ^1^ Department of Surgery, College of Medicine, Hawler Medical University, Erbil, Kurdistan Region, Iraq, hmu.edu.iq

**Keywords:** benign follicular nodular disease of the thyroid, fine-needle aspiration cytology, thyroid cancer, total thyroidectomy

## Abstract

**Background:**

Goiter is the most prevalent endocrine illness, and it is considered endemic when > 10% of a community has it. The patient may not notice the nodules, which typically form early in endemic goiter and later in sporadic goiter.

**Objectives:**

The objective of this study is to determine the incidence, histopathological subtypes, and risk of thyroid malignancy in patients with goiter, and to evaluate the diagnostic performance of ACR TI‐RADS‐guided fine‐needle aspiration cytology (US‐FNAC) using the Bethesda System for Reporting Thyroid Cytopathology 2023.

**Patients and Methods:**

This prospective, cross‐sectional descriptive study recruited patients with goiter in the Rizgary Teaching Hospital, Erbil, Iraq, from January 2020 to December 2024. Clinical and sonographic examinations were performed to confirm the diagnosis. To reduce the possibility of malignancy in goiter, a preoperative UG‐FNAB was performed. Patients underwent surgery; biopsies were analyzed, and histopathological analysis of surgically removed thyroid tissue was performed to determine the goiter type, Bethesda category, and malignancy status.

**Results:**

Among 128 diagnosed patients with goiter, 76 (59.4%) were diagnosed with BFN, in which most of them were females (*n* = 63, 82.9%), with a median age of 36 years. UG‐FNAB indicated colloid goiter in 28 (36.74%) cases, follicular neoplasm/suggestive malignancy in three (3.94%) cases, malignant in seven (9.2%) cases, and inconclusive in 35 (46.1%) cases. Based on the Bethesda Classification (2023), FNAC results indicated that Bethesda Category I (nondiagnostic or unsatisfactory) was more commonly detected (*n* = 35, 46.1%), followed by Category II (benign) (*n* = 28, 36.8%), whereas Category III (atypia of undetermined significance) was not detected (*n* = 0, 0%). Categories IV (follicular neoplasm or suspicious follicular neoplasm) and V (suspicious for malignancy) were found in three cases each (3.94% each), and Category VI (malignant) in seven cases (9.2%). The histopathological analysis found 15 (19.7%) individuals had a TC, including papillary carcinomas (*n* = 8, 53.3%), follicular carcinomas (*n* = 4, 26.6%), Hurthle cell carcinomas (*n* = 2, 13.3%), and anaplastic carcinoma (*n* = 1, 6.6%). Additionally, patients with BFN were 3.35 times more likely to develop thyroid cancer than those with a solitary thyroid nodule.

**Conclusions:**

The incidence of goiter is slightly high among patients in this locality, especially median‐aged females. However, the incidence of TC is low when successfully treated with total thyroidectomy.

## 1. Introduction

The term goiter refers to an enlargement of the thyroid gland that can be toxic or nontoxic, diffuse or nodular, and single or numerous. An enlarged thyroid gland with several areas of nodularity is called “multi” [1]. The most prevalent form of endocrine diseases is the nodular disease of the thyroid, which affects 500–600 million people worldwide [2]. Women and older people have an increased risk of developing nonendemic goiter [3]. The development of goiter in endemic areas is attributed to iodine deficiency and sociodemographic factors [4]. Although the patient may not notice the goiter until their 40s or 50s in endemic goiter, nodules develop earlier than sporadic goiter [5].

Goiter is characterized by the presence of thyroid nodules (dominant focal structural lesions) without biochemical abnormalities of the thyroid gland. It has been reported that the rate of autopsy identification of incidental thyroid cancer (ITC) has been progressively increasing, which might be due to the 50% detection rate of nodules found in the thyroid autopsy checking [6]. In asymptomatic patients, a high‐frequency (10–15 MHz) linear ultrasound (U/S) probe detects thyroid nodules in 13% of patients, with a 29% rate of malignancy in these nodules [7]. It is not uncommon to find TC on histopathological examination of goiter samples either by tru‐cut biopsy or surgery, making a predicament in the decision on the type/extent of surgery and the existence of ITC that necessitates extra work and multiteam care [8]. Historically, goiter was assumed to pose a lower risk of cancer than a single thyroid nodule [9]. However, studies show that BFN has an incidence of malignancy anywhere from 7% to 17% [10]. Papillary carcinoma (PTC) has been reported as the common type of malignancy [11]. Fine‐needle aspiration cytology (FNAC) has improved the therapy of a single lesion in the thyroid, whereas in goiter, the malignant lesion cannot be differentiated from a benign; clinically or radiologically [12]. TC manifests as a solid, quickly expanding, painless nodule that is either self‐reported by the patient or detected through a clinical examination, in which confirmed FNAC can do diagnosis under US guidance [13]. TC rates vary globally from 0.9% to 13% [14]. An increase in the prevalence of TC may be caused by radiation exposure and the existence of modern tools for the detection of cancer [15].

For goiter, total thyroidectomy (TT) has been recommended in some cases, particularly in iodine‐deficient regions. The higher frequency of ITC in BFN makes sub‐TT a less‐than‐ideal treatment option. Long–term follow‐ups also show a significant risk of 50% recurrence following the surgery [16]. Therefore, this study is aimed at determining the incidence, histopathological subtypes, and risk of thyroid malignancy in patients with goiter, and at evaluating the diagnostic performance of US‐FNAC.

## 2. Patients and Methods

### 2.1. Study Design and Setting

This prospective, cross‐sectional descriptive study recruited patients with goiter in the Department of Surgery, Rizgary Teaching Hospital, Erbil, Iraq, from January 2020 to December 2024.

### 2.2. Inclusion Criteria

Patients with goiter, including euthyroidism and bilaterally localized lesions, were included.

### 2.3. Exclusion Criteria

**Figure 1:**
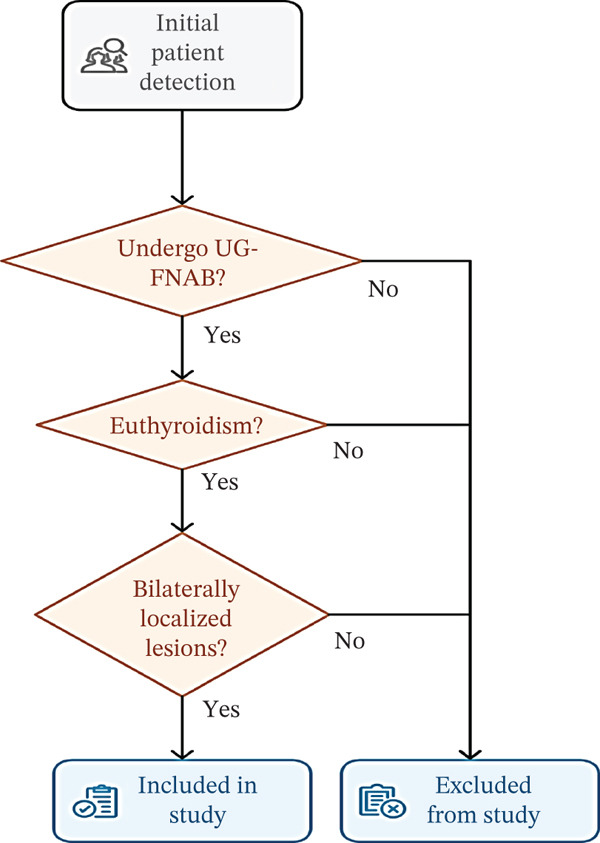
Showing patient selection process.

Patients with hyperthyroidism were excluded (Graves′ disease/toxic goiter) (Figure 1).

### 2.4. Study Protocol

Patients with goiter had been detected by clinical investigation (thyroid swelling, lymphadenopathy, thyroid hormone test, or assessment of vocal cord mobility by laryngoscope), ultrasonography, and/or ultrasound–guided fine‐needle aspiration biopsy (UG‐FNAB) on the dominant or chosen tumor to reduce the likelihood of malignancy; using linear probes operating between 7.5 and 10 MHz. Ultrasound features were evaluated and categorized according to the American College of Radiology (ACR) TI‐RADS system to determine nodule selection for FNAC, whereas cytological interpretation followed the Bethesda System for Reporting Thyroid Cytopathology (TBSRTC) 2023 (Categories I–VI) [17]. Moreover, their sociodemographic data (age and gender) were collected. When an anesthesiologist has finished examining a patient, the antibiotic is given as a single dose in the operating room immediately before the operation. Due to the patient′s compressive symptoms and cosmoses, as well as the diagnostic workup′s suspicions and mixed results, surgery was recommended as a therapy option. A TT was performed under general anesthesia after locating and protecting the recurrent laryngeal nerves and parathyroid glands. Then, surgically removed thyroid tissues were sent for histopathological analysis using the World Health Organization (WHO) classification of TC 2017 [18]. Patients were followed up according to the National Comprehensive Cancer Network (NCCN) [19]. Finally, diagnostic accuracy was performed using final histopathology as the reference standard. Whereas the risk of malignancy (BFN vs. solitary thyroid nodule [STN]) was also calculated, and the risk ratio (RR) was determined. Then, preoperative, intraoperative, and postoperative data were meticulously documented and standardized, and the outcomes were assessed.

### 2.5. Data Analysis

The data were analyzed using the Statistical Package for Social Science (SPSS, IBM, Chicago, United States, Version 26).

## 3. Results

After thorough examination using various techniques, it was detected that among 128 randomly diagnosed patients with goiter, 76 (59.4%) were diagnosed with BFN. Ultrasonography and TI‐RADS categorization revealed that 72 out of 76 patients (94.7%) had nodules requiring further evaluation (as an initial complaint), with a nodule size of 0.4–2.8 cm and an average diameter of 1.6 cm. These features, including microcalcifications and hypoechogenicity, were consistent with high–risk TI‐RADS categories (TR4 and TR5) in 29 (38.1%) patients. Additionally, most patients were females (*n* = 63, 82.9%), aged 31–40 years old (*n* = 27, 35.52%) with a median age of 36 years, a mean age of 34 ± 3.35 years, and an age range of 23–74 years (Table 1).

**Table 1:** Sociodemographic distribution of the patients with nontoxic multinodular goiter.

Variable		Number (percentage)
Gender	Male	13 (17.1)
	Female	63 (82.9)
Age group (years)	21–30	13 (17.1)
	31–40	27 (35.52)
	41–50	22 (28.9)
	51–60	9 (11.8)
	61–70	4 (5.2)
	> 70	1 (1.3)
Total	**76 (100)**	

The FNAC results according to TBSRTC 2023 revealed Bethesda Category I (nondiagnostic or unsatisfactory) in 35 (46.1%) cases, Category II (benign) in 28 (36.8%) cases, Category III (atypia of undetermined significance) were not detected, Category IV (follicular neoplasm or suspicious follicular neoplasm) in three (3.94%) cases, Category V (suspicious for malignancy) in three (3.94%) cases, and Category VI (malignant) in seven (9.2%) cases. Histopathological results revealed that 15 (19.7%) individuals had a TC, of which PTC (*n* = 8, 53.3%) were predominant, followed by follicular carcinomas (FTCs; *n* = 4, 26.6%), then Hurthle cell carcinomas (*n* = 2, 13.3%), and anaplastic carcinoma (ATC; *n* = 1, 6.6%) (Table 2).

**Table 2:** Correlation between FNAC (Bethesda system 2023) and final histopathology results.

Bethesda category	Total FNAC casesno. (%)	Final postoperative histopathology results
I: Nondiagnostic or unsatisfactory	35 (46.1)	Papillary CA: one, follicular CA: two
II: Benign	28 (36.8)	Papillary CA: one, follicular CA: one
III: Atypia of undetermined significance	0 (0.0)	—
IV: Follicular neoplasm or suspicious for follicular neoplasm	3 (3.9)	Follicular CA: one
V: Suspicious for malignancy	3 (3.9)	Papillary CA: one, Hurthle cell CA: one
VI: Malignant	7 (9.2)	Papillary CA: five, Hurthle cell CA: one, anaplastic CA: one
Total	76 (100)	Papillary CA: eight; follicular CA: four; Hurthle cell CA: two; medullary CA: zero; anaplastic CA: one; lymphoma CA: zero; poorly differentiated: zero

Abbreviation: FNAC, fine‐needle aspiration cytology.

Additionally, the correlation between FNAC and its final histopathological diagnosis revealed a sensitivity of FNAC in the detection of thyroid malignancy as 60% and specificity as 98.4%, whereas positive predictive value was 90% and the negative predictive value was 90.9%. FNAC shows a relatively high (40%) false‐negative rate despite its high specificity (Table 3).

**Table 3:** Fine‐needle aspiration cytology (FNAC) diagnostic performance.

Parameter	Value
Sensitivity	9/(9 + 6) = 60.0*%*
Specificity	60/(60 + 1) = 98.4*%*
Positive predictive value (PPV)	9/(9 + 1) = 90.0*%*
Negative predictive value (NPV)	60/(60 + 6) = 90.9*%*
False‐negative rate	40.0%

Finally, the risk of malignancy was compared between patients with goiter and those with STN, as among 76 patients with BFN, 15 patients were diagnosed with thyroid malignancy (19.7%), whereas only three out of 51 patients with STN were diagnosed with malignancy (5.9%) (Table 4). Patients with BFN were 3.35 times more likely to develop thyroid cancer than patients with STN, as shown in Table 4 and the following calculation:

RR=1576/351/=0.1970.059=3.35

**Table 4:** The risk of malignancy (benign follicular nodule vs. solitary thyroid nodule).

Nodule type	Malignant (number)	Benign (number)	Total (number)	Malignancy rate (%)
Multiple nodules (BFN)	15	61	76	19.7%
Solitary thyroid nodule (STN)	3	48	51	5.9%
Total	18	109	127	14.2%

*Note:* Risk ratio (RR) = 3.35; 95% CI [1.03, 10.89]; and *p* = 0.03.

## 4. Discussion

The etiology of goiter is multifactorial, mainly including iodine deficiency (initially established as a simple goiter), genetic predisposition, goitrogens, exposure to ionizing radiation, and thyroiditis. Consequently, this study is aimed at determining the incidence and types of TC in patients with BFN, and out of 128 patients with goiter, more than half of them (59.4%) were diagnosed with BFN using ultrasonography. Microcalcifications, irregular borders, increased vascularity, and hypoechoic appearance are ultrasonographic features associated with a marked risk for cancer, making TI‐RADS–based ultrasound assessment a critical tool for identifying suspicious nodules in patients with goiter [20]. The number of nodules does not alter the risk of a nodule being malignant, although the chance of cancer development increases in patients with multiple nodules. This could be due to various pathogens in the multiple nodules that may explain why FNA does not give an accurate diagnosis and increases the risk of malignancy in patients [21]. Regarding the sociodemographic data, females were predominant (82.9%) with a mean age of 34 ± 3.35 years. In this regard, a study in Iran also found female predominance among goiter patients (91.1%) with a mean age of 47.1 ± 12.9 years [22].

Because of the high false‐negative results of FNAC, the clinician needs to use the clinical and imaging data to improve the accuracy of TI‐RADS–guided UG‐FNAC [23]. Accordingly, in this study, UG‐FNAB was nondiagnostic in most cases (Category I, 46.1%), whereas Category II (benign) was indicated in 36.84% of cases. However, Categories VI (malignant), IV (follicular neoplasm or suspicious for follicular neoplasm), and V (suspicious for malignancy) were found in 17% of cases. Another study reported that FNAC results were negative for malignancy in 41.6% of cases, indeterminate in 53.5%, and suspicious in 5.0% of cases [22]. These variations might stem from divergent lifestyles, associated risk factors, and variations in sample sizes. Chieng et al. mentioned a low probability of malignancy in sonographically benign nodules with initial nondiagnostic FNAC results [24].

All patients in this study (*n* = 76) underwent TT, which is similar to Kabbash et al., who also performed TT as a rule of treatment for goiter patients [25]. Musbah et al. also recommend TT for all cases of goiter to reduce the risk of recurrence and development of malignancy in residual thyroid tissue and to prevent secondary thyrotoxicosis [26]. Among endocrine tumors, TC is the most common type with marked female predominance, and it is much higher in a single thyroid nodule than in multiple nodules [27]. The TC is rare in the pediatric age group; however, the rate increases with age [28]. The incidence of TC is about one to three cases in every 100,000 populations. Most goiters are harmless, but there is always a chance of cancer in iodine‐deficient locations, particularly among women [25]. In this study, 19.7% of patients had a TC, potentially higher than that found by Kalizewski et al. (2.12%) [29]. These variations might be related to the family history of TC and past neck exposure to radiation, which makes BFN doubtful for malignancy.

Polyclonal and monoclonal nodules coexist in BFN, indicating several distinct pathogenetic mechanisms as opposed to a single, consistent process. Cancer can develop even in clinically goiter because monoclonal nodules are true neoplastic proliferations, whereas polyclonal nodules result from hyperplastic responses. Kopp et al. initially illustrated this idea by displaying diverse clonal patterns within a single thyroid gland [30].

Among the detected TC in the current study, PTC was more common (53.3%), followed by FTC (26.6%), then Hurthle cell carcinomas (13.3%), and ATC (6.6%). The WHO regards papillary microcarcinoma as PTC when it is > 10 mm, which is frequently found incidentally in many postmortem autopsies [31], and if untreated, it becomes clinically evident [32].

Although TI‐RADS–guided U/S‐FNAC remains the cornerstone of preoperative evaluation of thyroid nodules, its diagnostic accuracy in BFN is limited. In the present study, FNAC showed excellent specificity but only moderate sensitivity, with a false‐negative rate of 40%. This finding is consistent with Bethesda 2023 benchmarks, which acknowledge reduced sensitivity in goiter due to sampling error and intraglandular heterogeneity. The coexistence of polyclonal hyperplastic nodules with monoclonal neoplastic foci may explain why malignancy can be missed despite benign or nondiagnostic cytology, thereby supporting the rationale for definitive surgical management in selected patients with goiter [30].

The inclusion of a control group with STN significantly strengthens the present study. Contrary to the traditional belief that multinodularity confers a lower cancer risk, our findings demonstrate that patients with goiter have a markedly higher risk of malignancy compared with those with a STN. The observed 3.35‐fold increased risk supports emerging evidence that multinodularity does not exclude malignancy and may reflect the coexistence of polyclonal hyperplastic nodules with monoclonal neoplastic foci. These findings provide a strong rationale for considering definitive surgical management in selected patients with goiter, particularly when cytology is inconclusive or discordant with clinical and ultrasonographic features.

The limitations of this study were that it was a single‐centered study, had a small sample size, and did not follow‐up with the patients for a long duration to report the RG rate.

## 5. Conclusions

Although BFN is primarily benign, malignancy is a considerable risk that needs comprehensive preoperative workup, including UG‐FNAB and imaging, to guide surgical management and optimize outcomes. A TT offers a definitive treatment of BFN; it removes all potentially affected tissue and minimizes the risk of recurrence. Further multicenter studies are needed to evaluate TC risk in BFN, focusing on genetic factors, environmental influences, and long‐term patient outcomes. Preoperative UG‐FNAB should be the standard practice when assessing BFN. Implementing regular postoperative follow‐up protocols can aid in the early detection of RG. Clinical guidelines adapted to areas and populations with high prevalence rates may standardize care and enhance early diagnosis.

## Author Contributions

A.M.D.: conceptualization, methodology, data collection, data analysis, and writing of the original manuscript.

## Funding

No funding was received for this manuscript.

## Disclosure

A preprint of this manuscript has previously been published in the Research Square [31].

## Ethics Statement

The ethical committee of the College of Medicine, Hawler Medical University, Erbil, Iraq, approved the entire patient selection and data‐gathering process (No. 4/6 on January 25, 2020). Written informed consent was obtained from the patients for the performance of the surgical procedure and participation in the study. All procedures performed in the study followed the ethical standards of the institutional and national research committee, along with the 1964 Helsinki Declaration and its later amendments.

## Consent

The study does not include any details or images related to any person.

## Conflicts of Interest

The author declares no conflicts of interest.

## Data Availability

The data that support the findings of this study are available on request from the corresponding author. The data are not publicly available due to privacy or ethical restrictions.
